# Mapping Vulnerability: Structure, Cascades, and Resilience in the Global Railway Vans Trade Network

**DOI:** 10.3390/e28040421

**Published:** 2026-04-09

**Authors:** Lingyun Zhou, Langya Zhou, Weiwei Gong, Cheng Chen, Baojing Huang

**Affiliations:** Transportation and Economics Research Institute, China Academy of Railway Sciences Corporation Limited, Beijing 100081, China; zhoulingyun@rails.cn (L.Z.); lyzhou@rails.cn (L.Z.); chch@rail.cn (C.C.); baojinghuang@rails.cn (B.H.)

**Keywords:** supply chain vulnerability, trade network analysis, cascading failures, railway equipment, systemic risk

## Abstract

Global supply chains face increasing vulnerability to disruptions from geopolitical tensions, natural disasters, and demand shocks. The global trade network for railway vans, critical for transcontinental freight transport, remains understudied despite its foundational role in global logistics. This study addresses the gap in understanding how the railway vans trade network structure evolves and responds to different types of shocks, moving beyond static analyses to capture dynamic vulnerabilities. Using UN Comtrade data (2013–2024), multi-level network analysis examined structural evolution at macroscopic, mesoscopic, and microscopic scales. Three risk propagation models simulated supply disruption, demand shock, and cooperation disruption scenarios to assess systemic vulnerabilities. The network transformed from a polycentric to core-periphery structure, with China dominating exports (67 partners in 2024) and Germany leading European integration. Supply disruptions from Romania and Czechia affected up to 114 countries under low risk absorption capacity (α = 0.1), while demand shocks from the USA impacted 53 countries. The disruption of strategic trade links, such as China–Australia, triggered severe systemic risks. The systemic criticality of risk sources varies by shock type, requiring context-specific resilience strategies. The findings guide policymakers in identifying critical vulnerabilities and designing targeted interventions for enhancing supply chain resilience in infrastructure sectors.

## 1. Introduction

In an increasingly interconnected global economy, the resilience of supply chains has become a paramount concern for both policymakers and industry leaders. While extensive research has focused on the vulnerabilities within supply chains for finished goods, such as electronics and automobiles, the networks that supply the foundational equipment for global logistics remain comparatively under-examined. The global trade network for railway vans and wagons—fundamental infrastructure enabling transcontinental freight transport—represents one such critical yet overlooked system. As railway freight accounts for approximately 28% of global land-based cargo movement [1], the stability of this network directly affects the efficient flow of countless other goods, making its structural integrity and resilience to shocks a matter of considerable strategic importance. Disruptions in this sector, whether from production bottlenecks, abrupt trade policy changes, or sudden demand shifts, can cascade through interconnected supply chains, creating far-reaching impediments to global trade.

Recent global events have underscored these vulnerabilities. The COVID-19 pandemic exposed the fragility of just-in-time production systems [2], while the 2021 Suez Canal blockage demonstrated how single-point failures can paralyze global logistics [3]. Despite these wake-up calls, scholarly attention to the railway equipment supply network remains limited. Existing literature on trade networks, while vast, often exhibits two key limitations that this study aims to address. First, many analyses adopt a static perspective, examining the network’s structure at a single point in time [4,5]. This approach fails to capture the dynamic evolution of trade relationships and the shifting roles of key countries over time, which is essential for understanding long-term trends and emerging vulnerabilities. Second, traditional methods for identifying systemic risk often rely on static centrality measures, equating a country’s importance with its volume of trade or number of connections. Such approaches overlook the complex contagion dynamics that characterize modern supply networks, as a nation’s true systemic risk profile may be more accurately defined by its potential to generate or propagate cascading failures—a dynamic property that static metrics cannot fully capture [6,7]. A critical gap therefore exists in understanding not just the structure of the railway vans trade network, but how its evolving architecture mediates the spread of different types of shocks.

Crucially, these gaps reflect a broader absence of resilience theory in infrastructure trade network research. Resilience theory, which examines systems’ capacity to withstand and adapt to shocks, provides a critical lens for understanding how railway van trade networks mediate disruption propagation. Yet, no study has yet integrated fundamental resilience principles and anti-risk capacity into the analysis of railway equipment trade networks. Recent advances have further deepened the understanding of resilience in complex networks. Proverbio and Boccaletti [8] offered a systematic perspective on the conceptual distinction and complementarity between robustness and resilience, arguing that networks maintaining efficiency under normal conditions may still exhibit poor recovery performance under targeted disruptions. Meanwhile, Hu et al. [9] demonstrated that adaptive and risk recovery strategies can significantly enhance supply chain resilience under cascading failures by integrating heterogeneous node capacities and continuous recovery mechanisms. Furthermore, Zelbi et al. [10] showed that topology-aware rewiring of supplier–customer relationships can reduce systemic risk by 16–50% without sacrificing production output. These advances suggest that infrastructure trade networks should be analyzed as adaptive systems in which structural concentration, failure propagation, and recovery potential jointly determine systemic vulnerability.

This study addresses this gap by providing a comprehensive, dual-perspective analysis of the global railway vans trade network from 2013 to 2024. The study’s primary contribution is its integration of a multi-level structural analysis with a suite of dynamic risk propagation models. First, the analysis maps the network’s evolution at the macroscopic, mesoscopic, and microscopic levels to identify significant shifts in its topology and the hierarchy of its key actors. Second, it advances beyond static analysis by simulating three distinct risk scenarios—supply disruption, demand shock, and cooperation disruption—to assess the network’s dynamic response to stress. This approach enables a more nuanced identification of critical vulnerabilities, revealing how the most systemically important countries and trade links change depending on the nature of the shock. Third, we explicitly link observed network dynamics to resilience theory, revealing how structural evolution creates both efficiencies and new vulnerabilities.

The empirical analysis yields several novel insights. The findings reveal that the network has transitioned towards a more centralized, core-periphery structure that, while potentially more efficient, is also prone to new forms of systemic risk. This structural evolution parallels theoretical predictions about network formation under globalization pressures [11,12], yet introduces unique vulnerabilities specific to the railway equipment sector. The results provide concrete, data-driven evidence that can inform more sophisticated and resilient supply chain strategies for both public and private sector stakeholders.

The remainder of this study is organized as follows: Section 2 reviews the relevant literature. Section 3 describes the methodology and data. Section 4 presents the results of the structural analysis. Section 5 details the findings from the risk propagation simulations. Section 6 discusses the implications of the findings and offers targeted policy suggestions. Finally, Section 7 concludes this study.

## 2. Literature Review

### 2.1. Topological Structure of Trade Networks

The study of trade networks has undergone a fundamental transformation in recent years, driven by unprecedented global disruptions and methodological innovations in network science. The COVID-19 pandemic and subsequent supply chain crises have catalyzed new theoretical frameworks that move beyond traditional static analyses. Importantly, Antràs and Chor [13] reconceptualized global value chains through the lens of “relationship stickiness,” demonstrating that supply network resilience depends critically on the switching costs and relationship-specific investments that create path dependencies. Building on this framework, Bonadio et al. [14] utilized a quantitative trade model to show that one-quarter of the pandemic’s economic impact stemmed from supply chain disruptions rather than direct health effects, underscoring the amplification mechanisms inherent in interconnected production networks. These findings have profound implications for understanding sector-specific vulnerabilities, particularly in capital-intensive industries like railway equipment manufacturing where relationship-specific investments are substantial.

Recent advances in complex network theory have enabled more sophisticated analyses of cascading failures and systemic risk in trade systems. Ialongo et al. [15] introduced a groundbreaking methodology for reconstructing firm-level production networks from limited data, revealing that actual supply chains exhibit significantly higher clustering and modularity than previously thought. This methodological breakthrough enabled Taylor et al. [16] to demonstrate empirically that supply network topology follows neither pure scale-free nor random patterns but rather exhibits a hybrid structure with “nested vulnerability pockets” where localized shocks can trigger disproportionate systemic effects. Complementing these structural insights, Kashiwagi et al. [17] developed dynamic propagation models showing that the speed and extent of shock diffusion depend critically on the interaction between network topology and the heterogeneous capacities of nodes to absorb disruptions. However, these sophisticated models have primarily been applied to electronics and automotive sectors, leaving infrastructure equipment networks—which operate under fundamentally different temporal dynamics and replacement cycles—largely unexamined. More recently, Sobb and Turnbull [18] developed a cascading failure model for intelligent transport systems that demonstrates how failure pathways interact with system architecture and mission continuity, reinforcing the need for sector-specific vulnerability assessment. In the domain of transportation infrastructure, Dong et al. [19] showed that railway network resilience benefits from combining structural indicators with disturbance-specific performance and recovery metrics. Additionally, Ma et al. [20] analyzed the global aircraft trade network and confirmed that contraction and rising concentration significantly alter system robustness under targeted attacks. Together, these studies highlight the importance of extending cascading failure and resilience analysis to infrastructure equipment trade networks such as the railway vans sector.

### 2.2. Vulnerability of Trade Networks

The pursuit of greater supply chain resilience and security has driven significant reconfiguration in contemporary trade networks. Javorcik et al. [21] provided compelling evidence that firms have actively reorganized their supply chains along geopolitical lines since 2020, with strategies such as “near-shoring” and regional diversification fundamentally altering traditional efficiency-based network configurations. This trend is particularly pronounced in strategic sectors, as demonstrated by Goldberg and Reed [22] who documented a 40% increase in supply chain reconfigurations involving critical infrastructure components. Moreover, Kleinman et al. [23] employed a spatial equilibrium model to show that such strategic reorganizations, by deviating from economically optimal structures, generate substantial welfare losses—approximately 5% of global GDP. For the railway equipment sector, where technical standards and gauge compatibility create natural regional clusters, these strategic realignments may have particularly severe efficiency implications.

Environmental sustainability concerns have added another layer of complexity to trade network analysis, with recent studies revealing intricate relationships between network structure and carbon emissions. Zhu et al. [24] pioneered the integration of carbon accounting into trade network models, demonstrating that supply chain length and complexity directly correlate with embedded emissions. Their findings are particularly relevant for heavy industries, where Lin and Wang [25] showed that the iron and steel sector—a key input for railway equipment manufacturing—exhibits substantial regional variations in carbon intensity, with implications for the environmental impact of different supply chain configurations. Furthermore, Cabernard et al. [26] revealed through multi-regional input-output analysis that relocating production in carbon-intensive industries often leads to carbon leakage rather than genuine emission reductions, suggesting that network reconfiguration must consider global rather than local environmental impacts. These environmental considerations increasingly shape both regulatory frameworks and corporate procurement strategies, potentially driving fundamental reorganizations of railway equipment supply networks beyond what traditional economic efficiency models would predict.

Despite these theoretical and empirical advances, critical gaps remain in understanding how sector-specific characteristics shape network vulnerability and resilience. The railway equipment sector presents unique analytical challenges that generic trade models inadequately address: extreme asset longevity (often exceeding 30 years), high switching costs due to technical incompatibilities, concentrated production in few global centers, and strong government involvement in procurement decisions. Furthermore, Elliott et al. [27] highlighted that infrastructure equipment networks exhibit “criticality inversion,” where seemingly peripheral suppliers can become systemically important during crisis periods. Recent work by Pichler et al. [28] on the semiconductor shortage demonstrated that such inversions are particularly pronounced in sectors with limited substitutability and long production cycles. This study addresses these gaps by developing a tailored analytical framework that captures the railway equipment sector’s unique characteristics while incorporating recent methodological advances in dynamic risk propagation modeling. This direction is further supported by recent evidence: Hu et al. [9] demonstrated that combined adaptive and risk recovery strategies are especially effective in dense, highly interconnected supply networks under cascading failures, while Zelbi et al. [10] showed that topology-aware network rewiring can materially lower systemic risk without reducing output. These findings reinforce the need to analyze vulnerability in specialized trade structures not only as exposure to shocks, but also as the limited capacity of highly concentrated networks to reconfigure under stress. This study thereby contributes to both the theoretical understanding of sector-specific trade networks and the practical application of resilience strategies in critical infrastructure supply chains.

## 3. Methodology and Data

### 3.1. Data Description and Network Construction

The data for this study were sourced from the United Nations Comtrade Database (UN Comtrade), one of the most authoritative and comprehensive sources for global trade data. The research focuses on goods classified under the Harmonized System (HS) code 8606, which encompasses “Railway or tramway goods vans and wagons, not self-propelled.” To ensure the comprehensiveness and accuracy of the data, the analysis includes all major sub-categories within this classification: tank wagons (860610), self-discharging wagons (860630), covered and closed wagons (860691), open wagons (860692), and other unlisted wagons (860699). The selected time span is from 2013 to 2024, with data accessed on 2 September 2025, allowing for an examination of the structural evolution and risk characteristics of the trade network over this decade.

Based on this data, the global railway vans trade system is abstracted into a weighted directed network denoted as *G* = (*V*, *E*, *W*). In this model, V = {*v*_1_, *v*_2_, …, *v_k_*} represents the set of nodes, where each node *v_i_* corresponds to a participating country or region. The set of directed edges is represented by *E* ⊆ *V* × *V*. A directed edge (*v_i_*, *v_j_*) ∈ E exists from *v_i_* to *v_j_* if country *v_i_* exports railway vans to country *v_j_*, signifying a trade relationship between them. *W* is the set of weights corresponding to the edges in E, where a weight *w_ij_* quantifies the strength of the trade relationship by representing the total trade value (in US dollars) from the exporting country *v_i_* to the importing country *v_j_* within a specific year. Furthermore, the network’s topology can be described by an adjacency matrix A = [*e_ij_*], where *e_ij_* = 1 if a trade flow exists from *v_i_* to *v_j_*, and *e_ij_* = 0 otherwise. This network model provides a formal basis for the subsequent analysis of structural properties and risk propagation simulations.

### 3.2. Structural Properties of the Trade Network

The architecture of the global railway vans trade network is examined through a multi-level analytical framework. This approach allows for a comprehensive characterization of the network’s features at three distinct scales: (1) the macroscopic level, which assesses global properties such as connectivity patterns and overall topology; (2) the mesoscopic level, which identifies modular substructures or communities of densely interconnected countries; and (3) the microscopic level, which evaluates the roles and influence of individual countries within the trade system.

#### 3.2.1. Macroscopic Measures

At the macroscopic level, four key metrics are employed to characterize the network’s global structure.

Reciprocity measures the extent to which trade relationships are mutual. In a directed network, it is the proportion of edges that are bidirectional. A high reciprocity value indicates a prevalence of two-way trade partnerships. It is calculated as:

(1) $$R=\frac{L^{↔}}{L}$$

where $L^{↔}$ is the number of reciprocal edges and *L* is the total number of edges.

Degree Assortativity assesses the tendency of nodes to connect with other nodes of a similar degree. It is formally the Pearson correlation coefficient of the degrees at either end of an edge. For a directed network, this is typically measured between the out-degree of the source node and the in-degree of the target node. A positive assortativity coefficient (*a_s_* > 0) suggests that high-degree countries tend to trade with other high-degree countries, forming a “rich-club” structure. Conversely, a negative coefficient (*a_s_* < 0) indicates that high-degree countries are more likely to trade with low-degree ones. The formula is:

(2) $$a_{s}=\frac{\sum_{(i,j)\in E}\left(k_{i}^{\mathrm{out}\;}-\bar{k^{\mathrm{out}\;}}\right)\left(k_{j}^{\mathrm{in}\;}-\bar{k^{\mathrm{in}\;}}\right)}{\sqrt{\sum_{(i,j)\in E}{\left(k_{i}^{\mathrm{out}\;}-\bar{k^{\mathrm{out}\;}}\right)}^{2}\sqrt{\sum_{(i,j)\in E}{\left(k_{j}^{\mathrm{in}\;}-\bar{k^{\mathrm{in}\;}}\right)}^{2}}}}$$

where the sum is over all edges (*i*, *j*) in the set *E*, *k_i_^out^* is the out-degree of the source node *i*, *k_j_^in^* is the in-degree of the target node *j*, and $\bar{k^{\mathrm{out}\;}}$ and $\bar{k^{\mathrm{in}\;}}$ are the average out-degree and in-degree calculated over all edges.

Network Density quantifies the overall connectedness of the network by comparing the number of existing trade links to the total number of all possible links. A higher density suggests a more tightly integrated global market. The density of a directed network is given by:

(3) $$D=\frac{L}{N(N-1)}$$

where *L* is the number of actual edges and *N* is the number of nodes.

The Average Clustering Coefficient measures the local cohesiveness of the network. It reflects the probability that two trading partners of a given country are also trading with each other. A high average clustering coefficient points to the existence of tightly knit trading blocs or localized clusters. For a node *v_i_*, the local clustering coefficient *C_i_* is:

(4) $$C_{i}=\frac{2T_{i}}{k_{i}\left(k_{i}-1\right)}$$

where *T_i_* is the number of triangles involving node *v_i_* and *k_i_* is its degree. The network’s average is the mean of all *C_i_*.

#### 3.2.2. Mesoscopic Measures

To investigate the network’s intermediate-scale organization, community detection is employed to identify clusters of countries that are more densely traded among themselves than with the rest of the network. This study utilizes the Infomap algorithm, a method particularly well-suited for analyzing flow-based systems like trade networks. Infomap partitions the network by minimizing the map equation, which describes the theoretical limit for compressing information about a random walk on the network. The objective function is:

(5) $$L(M)=q_{↷}H(Q)+\sum_{i=1}^{m}p_{↺}^{i}H\left(P^{i}\right)$$

Here, *L*(*M*) is the description length of a network partition *M* into *m* modules. The first term, $q_{↷}H(Q)$, represents the entropy of movements between communities, while the second term, $\sum_{i=1}^{m}p_{↺}^{i}H\left(P^{i}\right)$, is the weighted average of the entropy of movements within communities. By minimizing *L*(*M*), the algorithm identifies a partition where the random walker tends to remain within the same community for extended periods, thus revealing the most significant modular structures in the trade flow.

#### 3.2.3. Microscopic Measures

Microscopic analysis focuses on node-level metrics to quantify the specific roles and importance of individual countries.

Degree Centrality is a fundamental measure of a node’s connectivity. In a directed network, it is decomposed into:

- In-degree (*k_i_^in^*): The number of incoming edges to node *v_i_*, representing the number of countries from which it imports.
- Out-degree (*k_i_^out^*): The number of outgoing edges from node *v_i_*, representing the number of countries to which it exports. The total degree is the sum *k_i_* = *k_i_^in^* + *k_i_^out^*. The formulas are:

(6) $$k_{i}^{\mathrm{in}\;}=\sum_{j\in V}e_{ji},\;k_{i}^{\mathrm{out}\;}=\sum_{j\in V}e_{ij}$$

Strength Centrality extends the concept of degree by incorporating edge weights (trade values), thus measuring a node’s total economic engagement. It is also decomposed into:

- In-strength (*s_i_^in^*): The sum of weights of all incoming edges, reflecting the total monetary value of a country’s imports.
- Out-strength (*s_i_^in^*): The sum of weights of all outgoing edges, reflecting the total monetary value of a country’s exports. These are calculated as:

(7) $$s_{i}^{\mathrm{in}\;}=\sum_{j\in V}w_{ji},\;s_{i}^{\mathrm{out}\;}=\sum_{j\in V}w_{ij}$$

### 3.3. Risk Propagation Models in the Trade Network

To investigate the vulnerability of the global railway vans trade network, this subsection develops a simulation framework based on the Cascading Failure Theory. The simulation takes the weighted directed network *G* = (*V*, *E*, *W*) constructed in Section 3.1 as the input structure. We simulate how localized shocks—whether from production failures, demand contractions, or relationship ruptures—propagate through trade linkages governed by the nodes’ risk absorption capacity (*α*) and the shock intensity (*p*). The framework comprises three distinct scenarios: supply disruption, demand shock, and trade relationship disruption.

#### 3.3.1. Supply Disruption Risk Propagation

The supply disruption scenario simulates a downstream cascade of failures initiated by a sudden drop in an exporting country’s production or export capacity. The process begins when a selected country *v_i_*, serving as the initial shock source, experiences a sudden decrease in its total export volume (out-strength) by a proportion *p*, causing its effective export value to drop from *s_i_^out^* to *s_i_^out^*^′^ = (1 − *p*) *s_i_^out^*. This reduction in supply is then distributed among all its direct downstream trading partners in proportion to their original trade shares, such that any importing country *v_j_* experiences a reduction in Δ*w_ij_* = *p*·*w_ij_* from *v_i_*. Each downstream country possesses a capacity to withstand risk and mitigate propagated shocks, defined in this study as the risk absorption capacity (*α*). The parameter α∈[0,1] represents the proportion of supply loss a country can tolerate before its own functionality is compromised. A country *v_j_* is considered to have failed if the cumulative supply shock exceeds this capacity, causing its remaining effective supply to fall below the critical level defined by (1 − *α*) *s_j_^in^*. To rigorously evaluate the model’s robustness, we further perform a quantitative sensitivity analysis by continuously varying *α* and the initial shock intensity parameter *p* to observe their joint effects on the network’s disruption scale.

#### 3.3.2. Demand Shock Risk Propagation

Contrary to supply disruption, the demand shock scenario simulates an upstream chain reaction triggered by a sudden contraction in an importing country’s market demand. This model is initiated when a selected country *v_i_* experiences a drop in its total import demand by a proportion p, reducing its effective import value to *s_i_^in^*^′^ = (1 − *p*) *s_i_^in^*. This demand contraction is transmitted to all its upstream suppliers, where each exporting country *v_j_* faces a reduction in its export value to *v_i_* of Δ*w_ji_* = *p*·*w_ji_*. The failure of an upstream supplier *v_j_* occurs if this demand shock causes its total export value to fall below its risk threshold α, according to the condition *s_j_^out^* (*t*) < (1 − *α*) *s_j_^out^*. Upon failure, the supplier is assumed to halt all its own import activities to mitigate further losses, an action that propagates the demand shock further upstream to its own suppliers. The cascade terminates when no new countries fail in the network.

#### 3.3.3. Trade Relationship Disruption

This scenario simulates the network instability caused by an abrupt cessation of trade between two specific countries, an event simplified as a targeted “edge failure.” The process starts when a specific trade edge (*v_i_*, *v_j_*) is severed, making its trade value *w_ij_* zero. This event directly impacts both partners, but the model focuses on the downstream propagation from the perspective of the importing country *v_j_*, as this path more directly affects the global supply chain. The importer *v_j_* experiences a drop in its total in-strength due to the loss of supply from *v_i_*. The failure condition is identical to that in the supply disruption model: *v_j_* fails if its total import value falls below its risk threshold (1 − *α*) *s_j_^in^*. Once *v_j_* fails, the subsequent cascading process follows the same mechanism as the supply disruption model, propagating the risk downstream until the system reaches a stable state.

#### 3.3.4. Simulation Outputs and Evaluation Metrics for Systemic Criticality

To quantitatively evaluate the impact of shocks and identify the most critical risk sources within the network, we define two key output metrics for the simulation:

- Disrupted Scale (*S*): This metric quantifies the total number of countries that fall into a failure state (where remaining trade value drops below the threshold) after the cascade stabilizes. It serves as the primary indicator of systemic criticality; a risk source (country or link) that triggers a larger *S* is considered to possess higher systemic importance.
- Propagation Duration (*T*): This represents the number of iterative time steps required for the network to reach a new stable state. It reflects the persistence of the crisis and the speed of risk diffusion.

By analyzing *S* and *T* across different initialization targets, we can map the network’s vulnerability profile and pinpoint the specific nodes or edges whose failure would cause the most catastrophic systemic collapse.

## 4. Structural Characteristics of Global Railway Vans Trade Networks

### 4.1. Macroscopic Structure

**Figure 1:**
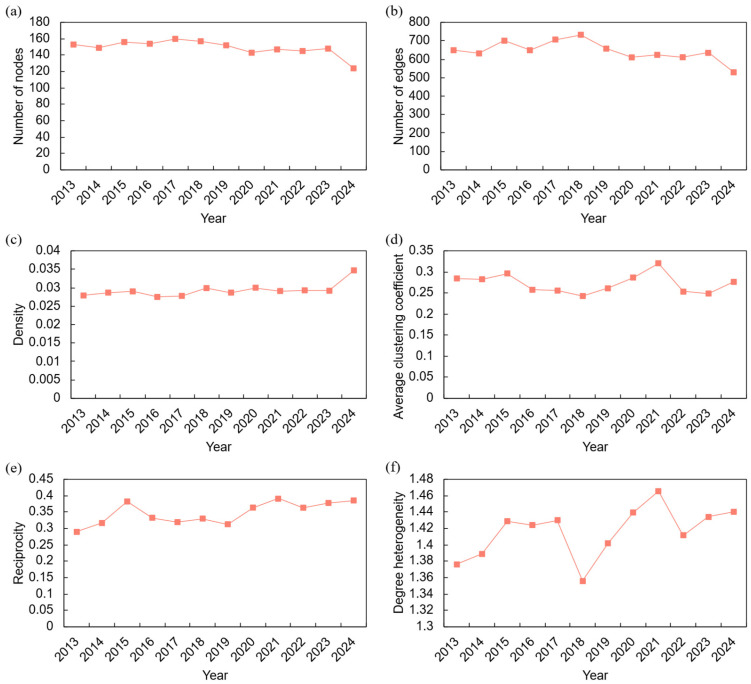
Macroscopic evolution of the trade network, 2013–2024. (**a**) Number of nodes; (**b**) Number of edges; (**c**) Network density; (**d**) Average clustering coefficient; (**e**) Reciprocity; (**f**) Degree heterogeneity.

An analysis of the network’s macroscopic evolution, presented in Figure 1, reveals several key structural trends over the 2013–2024 period. A systematic evaluation is provided below by examining the six indicators in three thematic groups: network scale, connectivity and clustering, and relational structure.

In terms of network scale, the number of active countries (Figure 1a) remained within a narrow band of 140 to 160 until 2018, with the number of trade links (Figure 1b) showing a commensurate trend between 550 and 750. After 2018, both indicators began a gradual decline, suggesting a systemic contraction that may be attributable to market consolidation, the exit of marginal participants, or a general reduction in international trade activity. The largely parallel trajectories of nodes and edges suggest that the network’s average connectivity per node remained relatively constant even as its overall size decreased.

Regarding connectivity and clustering, network density (Figure 1c) remained relatively stable, fluctuating between 0.025 and 0.035. In a shrinking network, this stability implies a slight increase in connectivity among the remaining participants. The average clustering coefficient (Figure 1d) displays a notable peak in 2020, reaching 0.32. This spike suggests a rise in localized, triangular trade relationships, possibly as a response to the COVID-19 pandemic, which may have encouraged countries to reinforce trade ties with their existing partners’ partners. The co-occurrence of stable density and elevated clustering in 2020 indicates that the network did not simply contract but underwent a structural tightening, with existing participants forming denser local clusters.

Turning to the relational structure, network reciprocity (Figure 1e) shows a persistent upward trend, increasing from approximately 0.3 to 0.38, indicating structural maturation characterized by an increasing prevalence of bidirectional trade over unilateral flows. In contrast, degree heterogeneity (Figure 1f) exhibited significant volatility. A sharp decline in 2018 suggests a potential disruption in the dominance of major trading hubs, possibly due to trade agreement realignments or national policy changes. The subsequent fluctuations indicate a dynamic interplay between the consolidation of trade around major hubs and the fragmentation of trade among smaller players. Notably, the concurrent rise in reciprocity and the fluctuation in heterogeneity suggest that, while trade relationships became more mutual, the distribution of trade partners across countries remained uneven and sensitive to external shocks.

Taken together, the six macroscopic indicators portray a network undergoing adaptive reconfiguration rather than simple decline. The system responded to external shocks through multiple mechanisms: enhancing local cohesion during periods of crisis (the 2020 clustering peak), rebalancing the influence of dominant actors (heterogeneity shifts), and evolving toward a more streamlined, reciprocal structure. By 2024, the network had become smaller in scale but denser, more reciprocal, and more tightly clustered—characteristics that suggest a trade-off between global breadth and structural robustness.

### 4.2. Mesoscopic Structure

**Figure 2:**
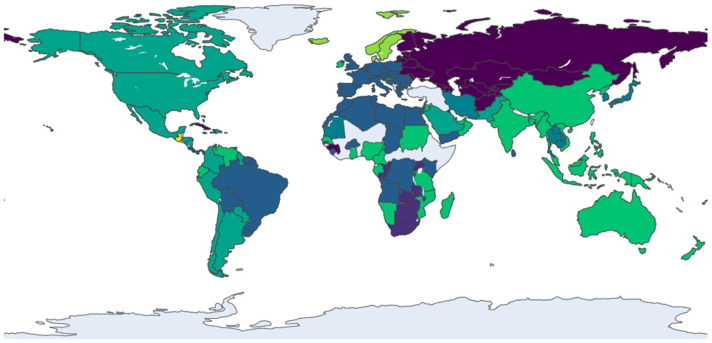
Community structure of the trade network in 2013. Note: Node colors represent distinct communities identified by the Infomap algorithm; color assignments are specific to this year and are not comparable across figures.

**Figure 3:**
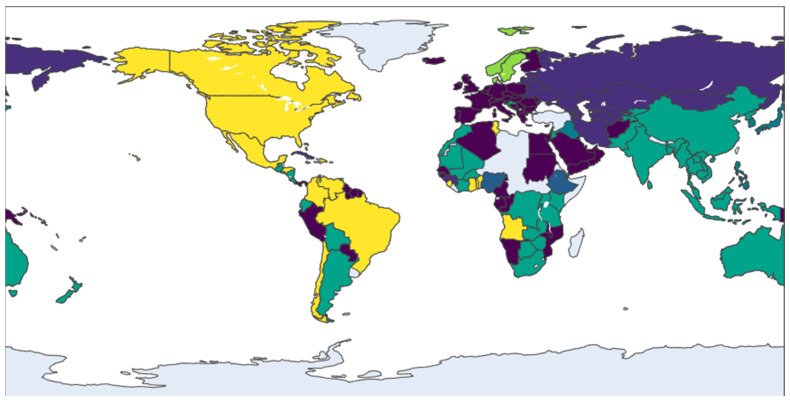
Community structure of the trade network in 2017. Note: see Figure 2.

**Figure 4:**
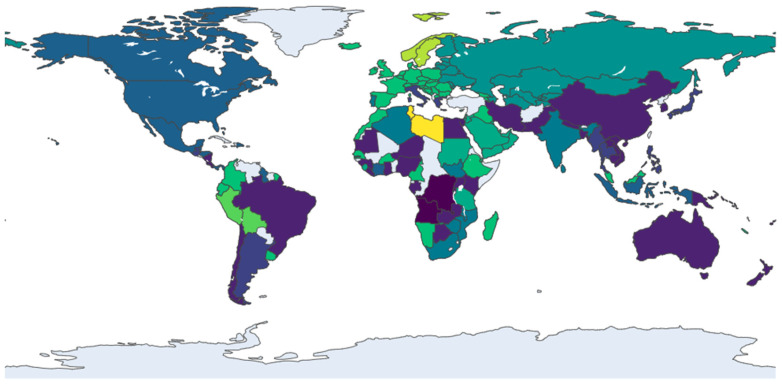
Community structure of the trade network in 2021. Note: see Figure 2.

**Figure 5:**
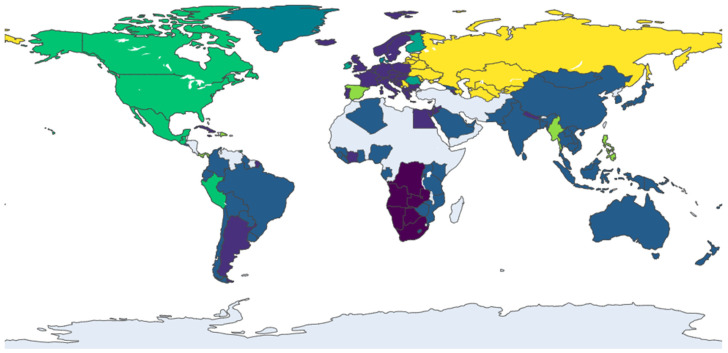
Community structure of the trade network in 2024. Note: see Figure 2.

At the mesoscopic level, an analysis of community structure reveals the dynamic formation and reconfiguration of trade blocs within the global railway vans network. Using the Infomap algorithm, distinct communities of densely interconnected countries were identified for the years 2013, 2017, 2021, and 2024, as visualized in Figure 2, Figure 3, Figure 4 and Figure 5. The evolution of these communities illustrates a clear structural transition from a regionally fragmented system to a more globalized, yet increasingly centralized, network. It should be noted that in each figure, node colors represent distinct communities identified by the Infomap algorithm for that specific year. Since the community detection is performed independently for each time snapshot, the same country may appear in different colors across figures, reflecting its changing community membership over time rather than any fixed categorical attribute.

The network’s evolution from 2013 to 2021 marks a decisive shift from a polycentric to a core-periphery structure. In 2013 (Figure 2), the system was characterized by distinct, geographically defined communities, such as an Asia-Pacific cluster (including Australia and Malaysia) and a dense European bloc (led by Germany and Italy), with countries like the USA serving as inter-regional bridges. By 2017 (Figure 3), this landscape had been fundamentally reshaped by the emergence of China as a dominant global hub, whose expansive connections began to integrate previously separate regions. This period also saw the European community, centered around Germany and France, strengthen its internal cohesion and solidify its role as a major core bloc. This trend toward centralization continued into 2021 (Figure 4), as new trade corridors linking the core regions with Central Asia and Africa became more prominent, and Eastern European nations like Romania and Serbia emerged as key secondary connectors, increasing the network’s overall integration.

In the most recent snapshot from 2024 (Figure 5), the network exhibits a dual trend of consolidation and diversification. The established hubs—notably China and the European bloc—remain central, though China’s connectivity has slightly decreased, suggesting a potential stabilization of its expansive growth. The most significant development is the increased integration of previously peripheral regions. Nations from Southeast Asia (e.g., Singapore, Thailand), South America (e.g., Brazil), and Africa (e.g., South Africa) have become more embedded in the network, indicating a deepening of globalization. The emergence of specialized secondary hubs, such as Spain and Saudi Arabia, further points to a more complex and multi-layered global system.

### 4.3. Microscopic Structure

A microscopic analysis of the network reveals the shifting hierarchy and specialized roles of individual countries. By examining centrality measures, which quantify both the number of trade connections (degree) and the monetary value of those connections (strength), it is possible to identify the key players and trace their evolving influence within the global railway vans trade system.

An analysis of degree centrality (Table 1) highlights the network’s key connectors. China consistently maintained its position as the most connected country over the decade, although its number of trade partners saw a slight decline from its peak. A stable European core, comprising Germany and France, also remained highly connected. A significant development is the rise in the Netherlands, which ascended from outside the top rankings to become one of the most central nodes by 2021, underscoring its growing importance as a logistical gateway. Meanwhile, the influence of other major players fluctuated; the USA held a steady but not dominant position, while the connectivity of some formerly prominent trading nations diminished, particularly after 2017, likely reflecting overarching economic and structural shifts. Decomposing degree into in-degree (import partners) and out-degree (export markets) reveals distinct functional roles (Table 2 and Table 3). China’s dominance is overwhelmingly driven by its vast number of export destinations (out-degree), cementing its role as the primary global supplier. In contrast, countries like the Netherlands and Germany consistently ranked high in in-degree, confirming their function as major import and distribution hubs within Europe. Other nations, such as France and the United Kingdom, demonstrated more balanced profiles, appearing prominently in both in-degree and out-degree rankings. The emergence of new countries like Malaysia and Spain in the top in-degree rankings by 2024 signals a diversification of supply chains and the growing integration of new economies into the network

While degree measures connectivity, strength centrality, which is based on trade value, reveals the network’s economic heavyweights. An analysis of total strength (Table 4) shows that the USA has been a pivotal player, with its total trade value consistently ranking at the top. A striking trend is the dramatic rise in Mexico, which evolved into the second-largest player by trade value in 2024, highlighting the deeply integrated North American trade bloc. European economies, particularly Germany and increasingly Slovakia, also solidified their positions as hubs of high-value trade. A disaggregated view of in-strength (import value) and out-strength (export value) further clarifies these economic roles (Table 5 and Table 6). The USA consistently functioned as the network’s primary sink for value, maintaining the top rank for in-strength throughout the period. Conversely, Mexico established itself as a dominant source of value, with its out-strength growing substantially to become the highest in the network by 2024. This dynamic underscore a powerful cross-border production system in North America. Within Europe, a similar regionalization is evident, with countries like Slovakia, Poland, and Romania emerging as significant exporters by value, often supplying the larger German market. These trends illustrate a complex global landscape where the immense import capacity of traditional economic powers like the USA fuels the export-oriented growth of both established and emerging industrial hubs.

## 5. Risk Simulation and Analysis of Global Railway Vans Trade Networks

Data for 2024 are used to simulate risk propagation in global railway vans trade networks. The simulation includes three realistic scenarios of the risk propagation model: demand disruption-driven, supply disruption driven, and intercountry cooperation disruption-driven risk propagation. The disrupted scale is the number of countries in a disrupted state when the risk propagation process stops. Additionally, the risk propagation duration is indicated by the iteration time.

### 5.1. Supply Disruption Risk Propagation Scenario

This subsection analyzes the network’s vulnerability to supply-side shocks, where an initial disruption in one country propagates downstream through the supply chain. The simulation results distinguish between two scenarios: a full disruption, where a failed country’s exports drop to zero, and a partial disruption, where exports are reduced by a proportion p.

**Figure 6:**
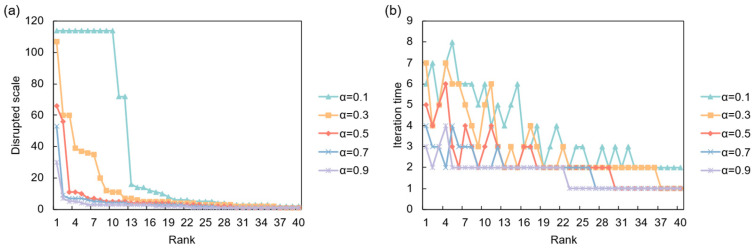
Network response to full supply disruption. (**a**) Disrupted scale (final number of failed entities) versus the initial shock’s risk ranking under different resilience levels (*α*); (**b**) Iteration time versus the initial shock’s risk ranking under different resilience levels (*α*).

**Figure 7:**
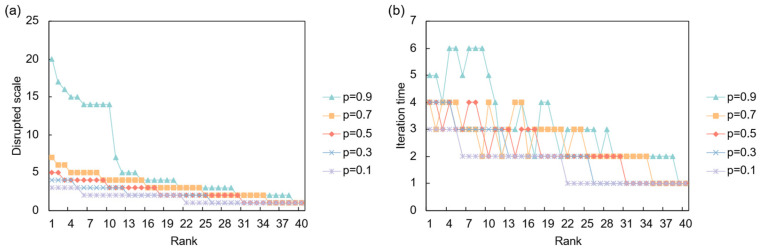
Network response to partial supply disruption. (**a**) Disrupted scale versus the initial shock’s risk ranking under different shock intensities (*p*); (**b**) Iteration time versus the initial shock’s risk ranking under different shock intensities (*p*).

The dynamics of these cascades are heavily influenced by the system’s intrinsic resilience, parameterized by the absorption capacity *α*. As illustrated in Figure 6, under a full disruption scenario, a low resilience (*α* = 0.1) leads to catastrophic, large-scale cascades, affecting over 100 countries when the shock originates from a high-ranking node. However, these cascades are relatively short-lived, stabilizing after a few iterations. Conversely, higher resilience (*α* ≥ 0.7) effectively suppresses propagation, with the disruption scale remaining minimal. This demonstrates a clear trade-off: while less resilient systems are prone to widespread failures, more resilient ones can contain shocks locally. Figure 7 shows that under a partial disruption, the impact is less severe but more persistent. Even with a high initial shock (*p* = 0.9), the total number of affected countries is significantly lower than in a full disruption. However, the system requires more iterations to stabilize, especially for intermediate values of *p*, suggesting that smaller, continuous disruptions can create prolonged instability.

An analysis of the most critical risk sources under full disruption reveals that a country’s systemic importance is highly dependent on the network’s overall resilience (Table 7). At a low resilience level (*α* = 0.1), the most critical nodes are highly integrated European countries (e.g., Romania, Czechia, Germany), where a single failure can trigger a massive cascade affecting nearly the entire network. As resilience increases (*α* = 0.3), the risk landscape shifts. While Germany remains a primary risk source, major global hubs like China and Japan emerge as critical, indicating that only disruptions from the largest players can overcome the system’s increased capacity to absorb shocks. At even higher resilience levels (*α* ≥ 0.5), the scale of disruptions diminishes dramatically, and the list of critical countries diversifies to include economies like the USA, Mexico, and South Africa, though their impact is far more contained. This progression underscores that as a system becomes more resilient, the focus of systemic risk shifts from regionally integrated players to globally central hubs.

**Figure 8:**
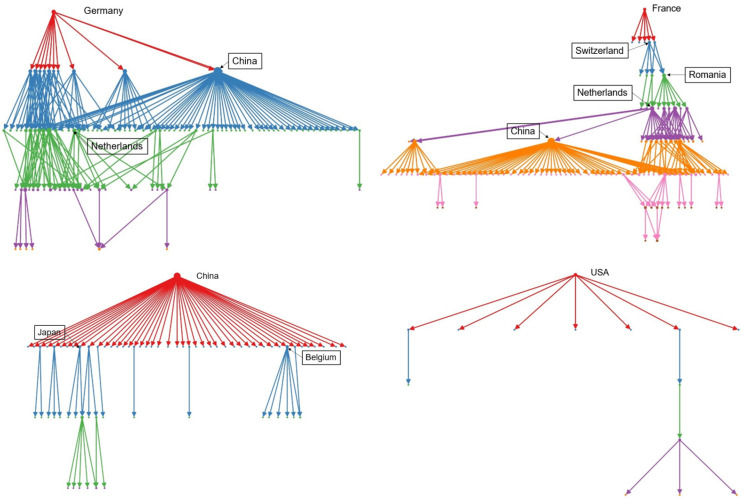
Supply disruption pathways for Germany, France, China, and the USA. Different colors represent distinct cascading propagation paths through downstream trading partners.

The specific pathways of these disruptions, visualized in Figure 8, highlight the unique structural roles of key economic powers. A shock originating in Germany, for instance, propagates through a dense, multi-layered web of European industrial dependencies, reflecting its role as the continent’s manufacturing core. A disruption in France follows a more tiered pattern, cascading through specialized, high-value connections to specific partners like Switzerland and Belgium. In contrast, a shock from China creates a broad, radial cascade, reflecting its position as a primary global exporter with a wide-reaching but less densely interconnected set of downstream partners. Finally, a disruption from the USA tends to be more localized, with fewer cascading steps, suggesting its impact is primarily concentrated on its immediate, high-value trade partners like those in the North American bloc. These distinct patterns reveal how each country’s unique position in the trade network shapes its potential to generate systemic risk.

**Figure 9:**
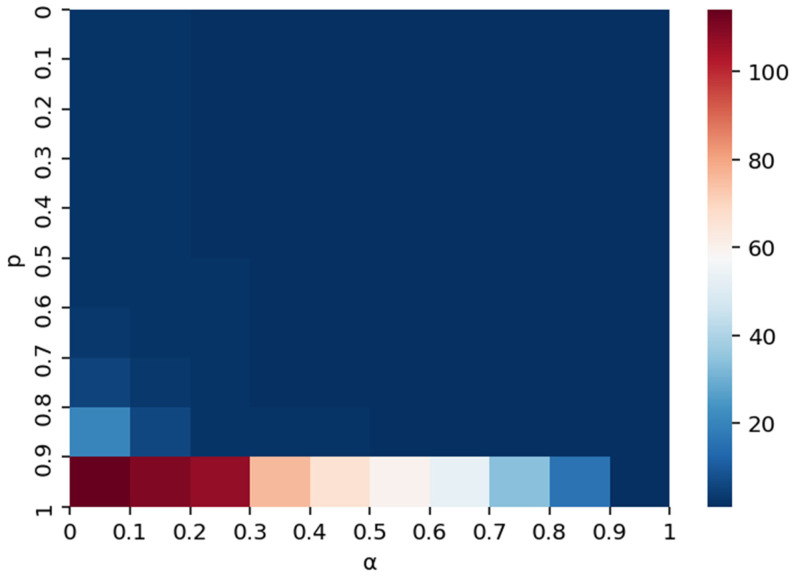
Effect of α and *p* on disrupted scale under supply disruption.

To rigorously validate the model’s robustness and move beyond discrete scenario testing, we conducted a comprehensive quantitative sensitivity analysis by continuously varying the risk absorption capacity (*α*) and the initial shock intensity (*p*) across their full theoretical intervals. Figure 9 presents the resulting heatmap, which illustrates the distribution of the maximum disrupted scale within this two-dimensional parameter space. In this visualization, color intensity corresponds to the magnitude of the maximum disrupted scale, with darker red hues indicating higher values—and thus greater systemic instability—while cooler blue tones denote smaller disrupted scales. The color bar provides a quantitative reference, ranging from 0 to 100, to facilitate the interpretation of these gradients.

From a systematic perspective, the quantitative assessment reveals distinct non-linear patterns regarding how risk absorption capacity (*α*) and shock intensity (*p*) interact to influence supply disruption propagation. Notably, the heatmap identifies a critical “phase transition” zone: larger red areas, representing elevated cascading failures, are concentrated in regions where the shock intensity *p* exceeds 0.9. In these high-intensity regimes, the system is fundamentally unstable; however, the analysis demonstrates that even moderate increments in risk absorption capacity (*α*) yield pronounced reductions in the disrupted scale, as evidenced by the rapid transition from deep red to lighter shades along the x-axis. This quantitative evidence suggests that when potential disruptions are severe (high *p*), enhancing the network’s intrinsic risk absorption capacity (*α*) serves as a highly effective lever for containing risk propagation and mitigating systemic vulnerability.

Conversely, when the shock intensity *p* remains below 0.9, the disrupted scale is comparatively constrained across the majority of the *α* spectrum. In these lower-intensity contexts, the marginal benefit of increasing risk absorption capacity is attenuated, as the baseline shock is insufficient to trigger extensive cascades regardless of the resistance threshold. This implies that substantial adjustments to *α* produce only slight diminutions in the disrupted scale, reflected in the subtle shifts from dark blue toward lighter blue tones. This asymmetry underscores the conditional role of risk absorption capacity as a control mechanism: its efficacy is largely contingent upon the severity of the initial shock. Specifically, *α* functions as a robust safeguard against catastrophic failures primarily when the system faces high-intensity shocks, whereas its capacity to further reduce disruption is limited in environments where the initial shock magnitude is already within a manageable range.

It is worth noting that the predominance of dark blue (low-disruption) areas in Figure 9 is not an artifact of the visualization method, but rather reflects the inherent robustness of the supply network under most parameter combinations. The concentration of high-disruption outcomes in a narrow corner of the parameter space visually confirms the threshold-dependent, “phase transition” nature of supply disruption cascades, where the system remains largely stable until both shock intensity and vulnerability simultaneously reach critical levels.

### 5.2. Demand Disruption Risk Propagation Scenario

This subsection shifts the focus to demand-side shocks. To address the distinction between structural and functional changes, we define the demand shock as a reduction in trade weight rather than a removal of topological links. The simulation is initiated when a target country experiences a contraction in its import orders. The magnitude of this contraction is controlled by the shock intensity parameter *p*.

In the partial disruption scenario, the trade network structure remains initially intact; the demand change is reflected solely by reducing the target node’s total import value (in-strength) by the proportion *p* (where $S_{in}^{\prime }=(1-p)S_{in}$). This reduction is then transmitted upstream to suppliers proportionally. In the full disruption scenario (*p* = 1), the demand drops to zero, simulating a complete market closure.

**Figure 10:**
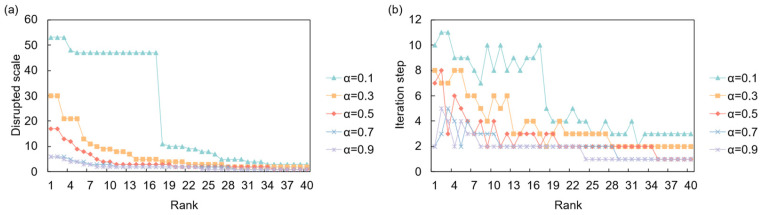
Network response to full demand disruption. (**a**) Disrupted scale versus the initial shock’s risk ranking under different resilience levels (*α*); (**b**) Iteration time versus the initial shock’s risk ranking under different resilience levels (*α*).

**Figure 11:**
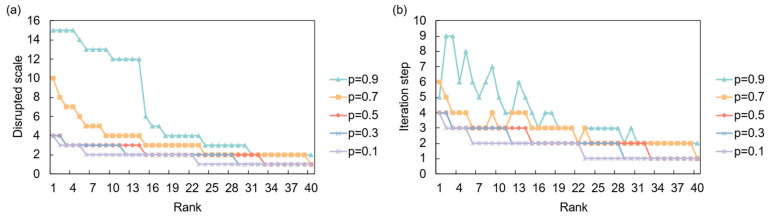
Network response to partial demand disruption. (**a**) Disrupted scale versus the initial shock’s risk ranking under different shock intensities (*p*); (**b**) Iteration time versus the initial shock’s risk ranking under different shock intensities (*p*).

The network’s response to a full demand shock is primarily governed by the systemic resilience parameter, *α*, as shown in Figure 10. In a system with low resilience (*α* = 0.1), a demand shock from a key importing nation can trigger a significant upstream cascade, affecting a large number of suppliers. As resilience increases, the network’s ability to absorb the shock improves dramatically, and the propagation is largely contained. This highlights that for demand-side risks, systemic resilience is crucial in preventing localized market contractions from escalating into widespread industrial downturns. In the partial disruption scenario (Figure 11), where the initial shock magnitude *p* is varied, the overall impact is less severe but can be more persistent. Higher values of *p* lead to a faster but more contained cascade, while lower values result in a slower, more prolonged period of instability, indicating that even minor but sustained demand contractions can have a lingering effect on supplier networks

An analysis of the most critical sources of demand risk under a full disruption scenario reveals a clear hierarchy of influential markets (Table 8). At low resilience levels (*α* = 0.1), the most systemically important nodes are major consumption and production hubs with extensive global supply chains, particularly the highly integrated North American economies (USA, Canada, Mexico) and core European nations (Germany, Austria). A demand collapse in these countries triggers a severe, widespread cascade. As the network’s resilience increases (*α* = 0.3 and higher), the scale of disruption shrinks, and the risk landscape diversifies. While the USA and Germany remain critical due to their central positions, other nations like the Philippines and Myanmar emerge as significant risk sources, likely reflecting their roles as key suppliers in specific vulnerable industries. This shift indicates that in a more resilient system, systemic risk is less about the absolute size of the import market and more about its strategic position within specific, critical supply chains.

**Figure 12:**
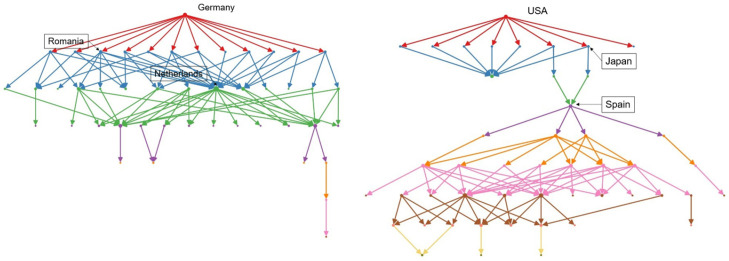
Demand disruption pathways for Germany and the USA. Different colors represent distinct cascading propagation paths through upstream suppliers.

The distinct propagation pathways originating from Germany and the USA, visualized in Figure 12, underscore their different roles as sources of demand risk. A demand shock in Germany propagates primarily through a dense, regionally clustered network of European suppliers, reflecting its position as the core of a continental industrial ecosystem. The impact is amplified within this bloc before diffusing globally. In contrast, a demand shock in the USA propagates through a more globally distributed, hub-and-spoke structure. The connections are more selective and strategic, linking the US market to key global suppliers like Japan and specialized European producers. This suggests that a US-based demand shock, while global in reach, would follow more targeted pathways, whereas a German shock would create a more concentrated, regional crisis.

**Figure 13:**
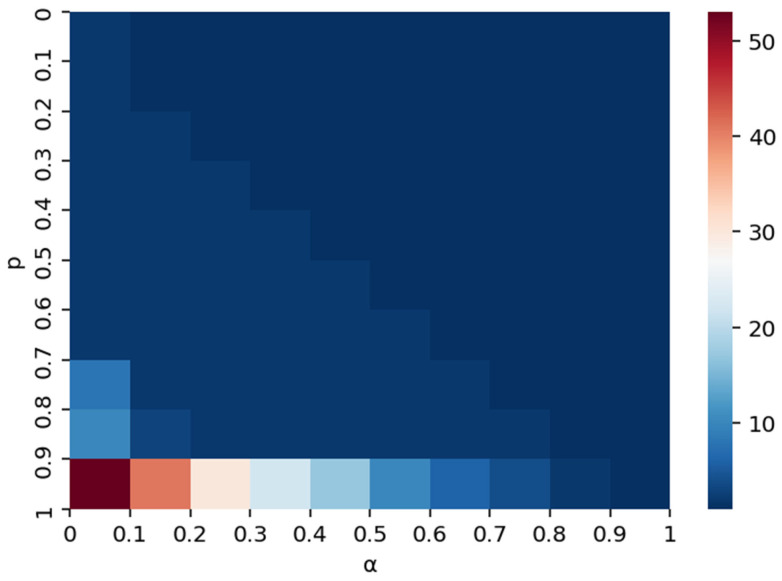
Effect of *α* and *p* on disrupted scale under demand disruption.

Figure 13 visualizes the interaction between demand shock intensity (*p*) and risk absorption capacity (*α*). Unlike supply disruptions, the demand-side risk shows a distinct sensitivity pattern. The “safe zone” (dark blue, indicating low disruption) covers a broader area, suggesting that the network is inherently more resilient to demand fluctuations than supply cuts. However, a critical tipping point is observed when *α* < 0.1. In this low-absorption region, even moderate demand shocks (p ≈ 0.5) can trigger significant upstream failures. This quantitative mapping highlights that while demand risks are generally containable, neglecting the baseline risk absorption capacity (*α*) can still lead to systemic fragility.

### 5.3. Cooperation Disruption Risk Propagation Scenario

This final scenario examines the impact of a targeted “edge failure,” where a specific bilateral trade relationship is severed due to non-market factors, such as sudden regulatory barriers or logistical breakdowns. This type of shock tests the network’s resilience to the loss of specific, strategic trade links.

**Figure 14:**
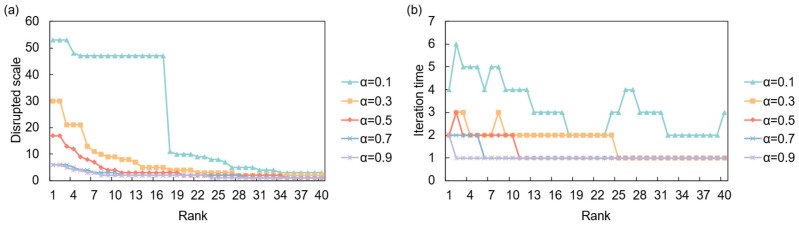
Network response to full cooperation disruption. (**a**) Disrupted scale versus the initial shock’s risk ranking under different resilience levels (α); (**b**) Iteration time versus the initial shock’s risk ranking under different resilience levels (α).

**Figure 15:**
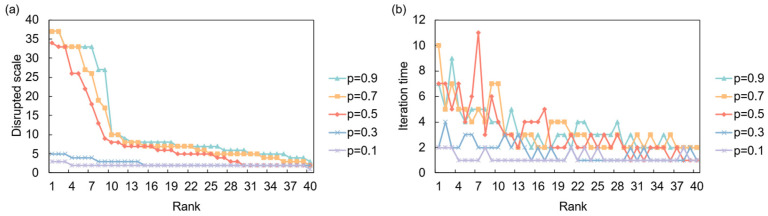
Network response to partial cooperation disruption. (**a**) Disrupted scale versus the initial shock’s risk ranking under different shock intensities (*p*); (**b**) Iteration time versus the initial shock’s risk ranking under different shock intensities (*p*).

The simulation results, presented in Figure 14 and Figure 15, demonstrate how the network responds to both full and partial cooperation disruptions. Under a full disruption (Figure 14), where a trade link is completely severed, the system’s response is highly dependent on its resilience (*α*). A low-resilience network experiences a sharp, severe cascade when a critical link is cut, though the disruption tends to stabilize relatively quickly. In contrast, a high-resilience network can effectively absorb the shock, with minimal propagation. In the partial disruption scenario (Figure 15), where trade flow is only reduced, the overall impact is less severe, but the recovery can be more complex. The system often requires more iterations to stabilize, particularly for mid-range disruption magnitudes, suggesting that a partial breakdown in cooperation can create prolonged, low-level instability that is harder to resolve than an acute, full-blown crisis.

An analysis of the most critical trade links under a full disruption scenario reveals the network’s key dependencies (Table 9). At a low resilience level (*α* = 0.1), the most vulnerable connections are those that bridge major economic blocs or connect core industrial hubs to essential resource suppliers. The China → Australia dyad emerges as the highest-risk edge, reflecting the strategic importance of this trade corridor. Additionally, connections within the European industrial core, such as those linking Bulgaria, Romania, and Poland to Germany, are highly critical, underscoring the region’s vulnerability to the failure of just a few key supply lines. As system resilience improves (*α* = 0.3), the risk landscape shifts. While the China → Australia link remains critical, its potential impact is significantly reduced. New transatlantic (Mexico USA) and other key intracontinental connections emerge as systemically important, indicating that in a more resilient network, risk is concentrated in the major global arteries of trade. The persistent appearance of Germany-centric edges across all resilience levels reinforces its role as Europe’s indispensable industrial anchor, where any disruption can have significant, albeit containable, regional consequences.

**Figure 16:**
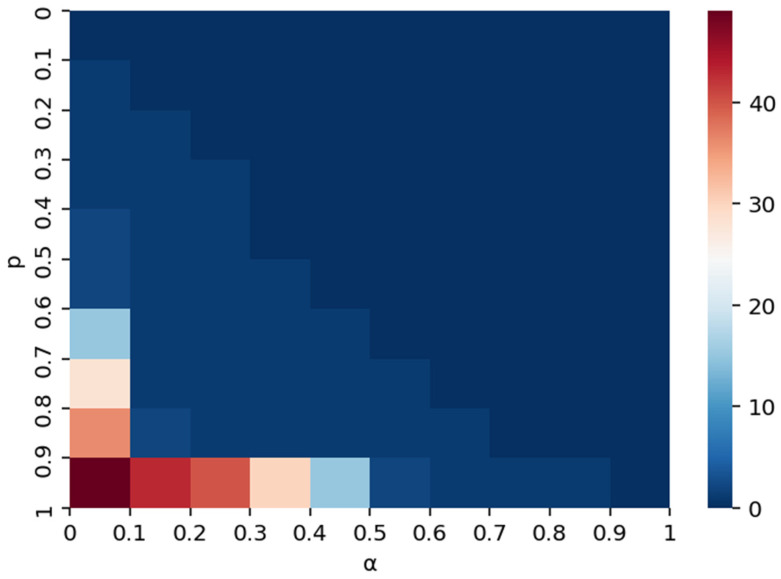
Effect of *α* and *p* on disrupted scale under cooperation disruption.

The interaction between relationship breakdown intensity (*p*) and risk absorption capacity (*α*) is depicted in Figure 16. The sensitivity analysis reveals a counter-intuitive phenomenon specific to cooperation disruptions: the system remains vulnerable even at lower shock intensities (*p* < 0.6) if *α* is not sufficiently high. The heatmap shows a diagonal boundary separating the stable and unstable states, indicating that as the severity of the relationship rupture increases (higher *p*), the requirement for risk absorption capacity (*α*) rises linearly to prevent cascading failures. This confirms that mitigating cooperation risks requires a dynamic approach to resilience building, proportional to the expected severity of trade disputes.

### 5.4. Model Validation

1. Validation against COVID-19 disruptions

The COVID-19 pandemic served as a profound stress test for global supply chains, exposing vulnerabilities across production, demand, and logistical coordination—all of which are directly relevant to our railway vans trade network. Empirical evidence from this period includes a documented ~12% decline in global trade volumes in 2020 (World Trade Organization, 2021) [29], driven by production halts (supply-side shocks), collapsing import demand (demand-side shocks), and border closures that disrupted cross-border trade flows (cooperation disruptions). Our simulation results align qualitatively with these observed dynamics. For instance, in the supply disruption scenario, simulations with low system resilience (α = 0.1) demonstrated catastrophic cascades affecting over 100 countries when a high-ranking node experienced a full export collapse. This mirrors the initial wave of disruptions in early 2020, when manufacturing shutdowns in China—a global hub for railway van production and transshipment—triggered ripple effects across industries reliant on these goods. As resilience parameters increased (α ≥ 0.7), the simulated disruptions were more localized and stabilized rapidly, paralleling the gradual recovery observed in 2021 as supply chains adapted to pandemic-related constraints. Similarly, the demand disruption scenario revealed that full import collapses in key markets generated upstream cascades in supplier networks, consistent with the sharp decline in railway freight volumes to Europe and North America during 2020. Partial demand shocks led to prolonged instability, reflecting the lingering effects of weak global demand throughout 2020–2021. Furthermore, the cooperation disruption scenario highlighted the vulnerability of trade flows to logistical bottlenecks, which were evident in the pandemic’s early months when cross-border customs clearance slowdowns and transport restrictions severely impacted railway operations.

2. Validation against Russia–Ukraine conflict

The Russia–Ukraine conflict introduced another critical test case, characterized by targeted sanctions, infrastructure damage, and the severing of key trade corridors—factors that directly align with our model’s cooperation disruption and supply disruption scenarios. Empirical data from this period indicates a ~40% drop in rail exports from Russia to Europe in 2022 (United Nations Conference on Trade and Development, 2022) [30], driven by sanctions that restricted the flow of critical goods and the physical disruption of rail links between Russia and its Western trading partners. Our simulations of full supply disruptions originating from high-exporting countries (e.g., Russia) produced large-scale cascades in neighboring regions, mirroring the real-world contraction of Russia–Europe rail trade. The disrupted scale and duration of these cascades were particularly pronounced under low-resilience conditions (α = 0.1), where the loss of a single critical trade link triggered widespread instability. This aligns with the observed immediate trade losses and logistical challenges reported in 2022, as European countries scrambled to find alternative routes for goods previously transported via Russian railways. The cooperation disruption scenario further validated these findings, demonstrating that the severing of specific bilateral trade links caused acute, high-impact disruptions. The prolonged recovery times observed in simulations for mid-range disruption magnitudes mirrored the ongoing challenges of reconfiguring trade routes and establishing new logistical partnerships in the conflict’s aftermath. Notably, the persistent criticality of high-exporting countries and strategic trade links in our simulations underscores their systemic vulnerability to geopolitical shocks, a pattern directly reflected in the conflict’s disruptive effects on global railway trade.

By comparing these simulation outcomes with empirical data, we observe a consistent qualitative alignment between modelled disruption dynamics and real-world crises. Specifically, the simulation’s identification of critical nodes (e.g., China, Germany, Russia) as sources of systemic risk, the cascading effects of supply and demand shocks, and the prolonged instability caused by cooperation disruptions all resonate with the documented impacts of COVID-19 and the Russia–Ukraine conflict. However, we acknowledge limitations in direct quantitative validation due to differences in data resolution, our model focuses on aggregated trade flows and topological metrics such as disrupted scale and iteration time, rather than absolute trade volume reductions. Additionally, sector-specific nuances are not fully captured in our generalized railway vans trade network. Nonetheless, the qualitative consistency between simulated and observed patterns—particularly in the roles of key countries, the propagation of shocks, and the influence of resilience parameters—strengthens the empirical credibility of our findings.

## 6. Discussion and Policy Suggestions

The findings reveal a system undergoing significant transformation, characterized by a shift towards a more centralized yet complex architecture. This concluding section discusses the primary implications of these findings and proposes targeted policy recommendations to enhance the resilience of this critical global supply chain.

### 6.1. The Double-Edged Sword of Globalization: Centralization and Vulnerability

The structural analysis presented in Section 4 reveals a fundamental shift in the trade network’s architecture over the past decade. The network has evolved from a polycentric structure with distinct regional blocs in 2013 to a more integrated, core-periphery system by 2024. This centralization, driven by the emergence of dominant hubs like China (as the primary exporter, Table 3) and the strengthening of the European industrial core (led by Germany, Table 1), likely reflects an increase in global economic efficiency. However, our findings suggest this efficiency comes at the cost of increased systemic vulnerability. The concentration of trade connections and value in a few key players means that localized shocks are now more likely to have global repercussions, a phenomenon not as pronounced in the more fragmented structure of the early 2010s.

This heightened risk is further evidenced by the network’s macroscopic properties. While the overall number of participants has slightly decreased, the persistent upward trend in reciprocity (Figure 1e) and the spike in clustering during the 2020 crisis (Figure 1d) indicate a system that responds to uncertainty by reinforcing ties within established communities. This creates denser, more tightly knit core groups. While beneficial for regional stability, it can amplify shocks within these blocs, as demonstrated by the massive cascading failures originating from European nations under low-resilience supply disruption scenarios (Table 7, *α* = 0.1). This finding aligns with the work of Acemoglu et al. [31], who argued that while microeconomic shocks may average out in highly dense networks, the failure of a few key nodes can trigger disproportionately large cascades. Our study provides empirical evidence of this in the context of a specific, critical industry. Recent theoretical advances by Baqaee and Farhi [32] further demonstrate that network centrality and productivity are complements in amplifying aggregate volatility, suggesting that the increasing dominance of China and Germany in the railway equipment network may paradoxically increase systemic fragility despite efficiency gains. This concern is reinforced by emerging evidence that robustness and resilience are not equivalent: networks that remain efficient under normal conditions may still exhibit poor recovery performance under targeted disruptions unless adaptive reconfiguration mechanisms are incorporated into system design [8,9,18].

### 6.2. Redefining Criticality: From Systemic Hubs to Strategic Links

A key contribution of this study is the nuanced identification of systemic risk sources, which challenges a one-size-fits-all definition of a “critical” country. The risk simulations in Section 5 demonstrate that a country’s systemic importance is highly context-dependent. Under supply disruption scenarios, the most critical nodes are often highly integrated regional players within dense industrial ecosystems, like Romania and Czechia (Table 7), whose failure triggers widespread regional collapse. Conversely, under demand disruption scenarios, the primary risk sources are the major global consumption hubs, namely the USA and its North American partners (Table 8), whose market contractions send shockwaves upstream across the globe. This distinction is crucial: policies aimed at mitigating supply risk should focus on the industrial cores of Europe and Asia, while policies for demand risk must address the economic health of North American and other major consumer markets. This context-dependency aligns with Hallegatte et al. [33], who demonstrated through agent-based modeling that the criticality of supply chain nodes varies dramatically with the type and duration of disruptions. This finding is also consistent with recent studies showing that node criticality depends strongly on disruption type, recovery regime, and local load-capacity asymmetry, rather than on static centrality alone [9,18].

Furthermore, the cooperation disruption analysis (Section 5.3) elevates the discussion from critical nodes to critical links. The finding that the severance of a single trade dyad, such as China → Australia or key intra-European links like Bulgaria → Germany (Table 9), can trigger significant cascades highlights a different dimension of vulnerability. This suggests that abrupt, non-market interruptions impacting just one strategic relationship could pose a systemic threat comparable to a major country’s internal failure. This result provides a quantitative underpinning for recent policy discussions on enhancing supply chain security and resilience [34], demonstrating that the most critical vulnerabilities may lie not within countries, but between them. The fact that the list of critical links changes with the system’s resilience level (*α*) further implies that risk is not static, but a dynamic property of the network’s overall health.

### 6.3. Policy Suggestions for Enhancing Supply Chain Resilience

The findings from this study lead to several specific, data-driven policy recommendations for both public and private sector stakeholders, moving beyond generic advice to offer targeted strategies.

First, for national governments and international bodies, a two-tiered approach to risk assessment is necessary.

- Identify and monitor context-specific risks: Policymakers should move away from a single list of “systemically important countries.” Instead, they should maintain distinct risk profiles based on different shock scenarios. For instance, an EU-level supply chain authority should pay special attention to the health of its Eastern European industrial suppliers (as identified in Table 7), while trade bodies like the WTO should monitor the demand stability of North American markets (Table 8) as a lead indicator for global industrial health. Furthermore, diplomatic and trade agencies should explicitly map and monitor the “critical trade edges” identified in Table 9, treating their stability as a matter of strategic importance. This recommendation aligns with Baldwin and Freeman [4], who argue for “supply chain stress tests” analogous to those used in banking regulation. This is further supported by recent research showing that resilience assessment should incorporate both cascading propagation and recovery behavior, and that policy interventions are more effective when they target specific structural bottlenecks rather than uniformly increasing redundancy across the entire network [9,10].
- Invest in systemic resilience (*α*): Our simulations consistently show that increasing the network’s absorption capacity (*α*) is the most effective way to mitigate all types of risk. In practical terms, this translates to policies that encourage diversification of suppliers and markets. For example, governments could offer tax incentives or subsidies for companies that can demonstrate a diversified sourcing strategy, reducing their reliance on any single country identified as a critical hub in our analysis. For critical import-dependent nations (Table 5), building up strategic reserves of essential goods can also directly increase their resilience threshold. Recent work by Grossman et al. [35] provides theoretical support for such policies, demonstrating that optimal trade policy in networked environments should account for supply chain resilience externalities.

Second, for corporations and industry consortiums, the insights can inform more sophisticated supply chain management.

- Conduct network-aware stress testing: Firms should use the methodologies outlined in this study to conduct stress tests on their own supply chains. A company sourcing from Germany, for example, should not only assess the risk of a German supplier failing but also the risk of that supplier’s own critical inputs from Romania or Poland being disrupted (as per the pathways in Figure 8). This multi-level view, informed by our community and risk propagation analysis, is more robust than traditional, linear supply chain risk assessments.
- Promote “smart” redundancy: Instead of simply diversifying to random new suppliers, firms can use our mesoscopic analysis (Section 4.2) to guide their choices. A “smart” redundancy strategy would involve establishing backup suppliers in different trade communities from the primary supplier. For a firm relying on a Chinese supplier (part of the dominant Asian bloc), establishing a secondary supplier in the Mexican or Eastern European blocs would provide greater resilience against large-scale, community-wide shocks than simply choosing another supplier within the same Asian bloc. This approach aligns with Son et al. [36], who found that firms strategically reduced network complexity by reconfiguring suppliers across geographic clusters after the 2011 Japanese earthquake. Similarly, Todo et al. [37] demonstrated that diversified supply networks outside disaster zones enabled faster production recovery, validating the benefits of cross-community redundancy over within-bloc diversification. Recent evidence further confirms that network rewiring and adaptive recovery can reduce systemic risk more effectively than undirected diversification alone; in particular, Zelbi et al. [10] demonstrated that topology-aware rewiring can lower systemic supply chain risk by 16–50%, while Hu et al. [9] showed that adaptive recovery strategies are especially effective when tailored to the heterogeneous capacities of firms within dense networks. Moreover, the network-aware analytical framework developed in this study can be extended to support AI-driven digital marketing and logistics decision-making in the railway freight sector. By integrating trade network intelligence with market monitoring and customer demand forecasting, railway logistics enterprises can develop more targeted and data-driven marketing strategies for diverse freight segments.

## 7. Conclusions

This study addressed the critical need for a more dynamic and nuanced understanding of risk within the global railway vans trade network, a foundational component of the world’s logistical infrastructure. By moving beyond traditional static analyses, this study integrated a decade-long examination of the network’s evolving structure with a multi-scenario simulation of cascading failures. The objective was to identify not only the most important actors within the system but also the specific structural configurations and shock types that pose the greatest systemic threat.

The analysis yielded two primary findings. First, the network’s structure has fundamentally transformed, evolving from a decentralized, regionally clustered system into a more globalized, core-periphery architecture centered on a few dominant hubs in Asia and Europe. Second, our risk simulations demonstrate conclusively that a country’s systemic importance is not a fixed attribute but is highly context-dependent. The sources of critical risk shift dramatically depending on whether the shock originates from a supply disruption (where integrated regional producers are key), a demand contraction (where major consumer markets are pivotal), or a targeted breakdown in cooperation (where specific strategic trade links become the primary vulnerability).

The principal takeaway from this study is that effective supply chain resilience strategies must be tailored to the specific nature of the risk being addressed. A one-size-fits-all approach that focuses solely on the largest or most connected countries is insufficient. Instead, policymakers and industry leaders must adopt a more sophisticated, network-aware perspective that recognizes the distinct patterns of vulnerability exposed by different types of shocks. By providing a framework to identify these context-specific risks, this study offers a concrete, data-driven foundation for building a more robust and resilient global trade ecosystem.

While this study provides a comprehensive analysis, its limitations open avenues for future research. The reliance on aggregated national-level trade data precludes an examination of firm-level dynamics, and the risk models, while insightful, do not account for real-time adaptive behaviors. Future work could address these gaps by developing multi-layer network models that incorporate related economic sectors or by employing agent-based modeling to simulate more complex, strategic responses to crises. Such research would further refine our understanding of supply chain vulnerabilities and contribute to the development of even more sophisticated resilience strategies. In particular, integrating the structural risk insights from trade network analysis with artificial intelligence-based logistics decision-support systems could enable more intelligent and precise marketing and operational strategies for railway freight enterprises.

## Figures and Tables

**Table 1:** Top 10 countries by degree centrality for selected years.

Rank	2013		2017	
	Country	Value	Country	Value
1	China	77	China	89
2	Germany	63	Germany	73
3	France	57	France	59
4	USA	44	Netherlands	45
5	Russian Federation	38	USA	43
6	Italy	37	United Kingdom	39
7	United Kingdom	36	Italy	36
8	Ukraine	28	Russian Federation	31
9	Spain	28	Poland	30
10	United Arab Emirates	26	Romania	27
**Rank**	**20** **21**		**202** **4**	
	**Country**	**Value**	**Country**	**Value**
1	China	77	China	70
2	Netherlands	64	Netherlands	60
3	Germany	60	Germany	55
4	France	55	France	41
5	United Kingdom	39	Italy	35
6	USA	39	Spain	34
7	Italy	37	USA	31
8	Russian Federation	32	United Kingdom	31
9	Slovakia	29	Czechia	30
10	Poland	26	Romania	29

**Table 2:** Top 10 countries by in-degree centrality for selected years.

Rank	2013		2017	
	Country	Value	Country	Value
1	Russian Federation	20	Germany	24
2	Germany	18	France	19
3	France	17	Netherlands	19
4	Italy	16	Sudan	19
5	United Arab Emirates	16	Russian Federation	15
6	Saudi Arabia	15	Poland	15
7	USA	14	Hungary	15
8	Ukraine	12	USA	13
9	Switzerland	11	Switzerland	13
10	Belgium	11	Czechia	13
**Rank**	**20** **21**		**202** **4**	
	**Country**	**Value**	**Country**	**Value**
1	Netherlands	28	Netherlands	30
2	Germany	24	Germany	21
3	France	18	France	18
4	United Kingdom	16	United Kingdom	17
5	Italy	15	USA	15
6	Russian Federation	14	Switzerland	15
7	Saudi Arabia	14	Italy	14
8	Austria	14	Romania	14
9	Slovakia	13	Malaysia	14
10	Ukraine	13	Spain	13

**Table 3:** Top 10 countries by out-degree centrality for selected years.

Rank	2013		2017	
	Country	Value	Country	Value
1	China	69	China	80
2	Germany	45	Germany	49
3	France	40	France	40
4	USA	30	USA	30
5	United Kingdom	26	United Kingdom	27
6	Spain	24	Netherlands	26
7	Italy	21	Italy	24
8	India	20	Spain	20
9	Russian Federation	18	Russian Federation	16
10	Ukraine	16	Romania	16
**Rank**	**20** **21**		**202** **4**	
	**Country**	**Value**	**Country**	**Value**
1	China	72	China	67
2	France	37	Germany	34
3	Netherlands	36	Netherlands	30
4	Germany	36	France	23
5	USA	28	Italy	21
6	United Kingdom	23	Spain	21
7	Italy	22	Czechia	18
8	Russian Federation	18	Poland	17
9	Slovakia	16	USA	16
10	Poland	15	Slovakia	16

**Table 4:** Top 10 countries by strength centrality for selected years.

Rank	2013		2017	
	Country	Value	Country	Value
1	USA	2,747,044,790	USA	3,311,973,735
2	Ukraine	2,731,576,934	Mexico	2,309,091,612
3	Russian Federation	2,357,782,383	Canada	1,035,671,971
4	Mexico	2,136,521,038	China	751,243,719
5	China	1,337,107,058	Germany	729,952,081
6	Australia	1,009,267,591	Russian Federation	678,852,087
7	Germany	857,710,175	Slovakia	590,824,852
8	Kazakhstan	762,106,191	Poland	427,194,900
9	Canada	653,798,187	Ukraine	300,272,504
10	Slovakia	401,773,997	Kazakhstan	250,164,575
**Rank**	**20** **21**		**202** **4**	
	**Country**	**Value**	**Country**	**Value**
1	USA	2,790,938,503	USA	4,764,788,874
2	Mexico	2,058,223,094	Mexico	4,079,297,797
3	Germany	1,000,805,018	Germany	1,373,498,167
4	Canada	978,943,724	Slovakia	1,015,751,677
5	Slovakia	918,407,276	Canada	790,690,355
6	Russian Federation	838,991,840	China	761,926,518
7	Poland	670,363,092	Romania	531,164,972
8	China	545,162,172	Poland	471,062,647
9	Australia	344,567,353	Australia	451,730,860
10	Kazakhstan	331,106,897	Bulgaria	337,050,979

**Table 5:** Top 10 countries by in-strength for selected years (2013, 2017, 2021, and 2024).

Rank	2013		2017	
	Country	Value	Country	Value
1	Russian Federation	2,017,024,423	USA	2,236,989,070
2	USA	1,921,951,004	Canada	927,478,146
3	Australia	1,007,987,684	Germany	586,266,104
4	Kazakhstan	746,007,514	Argentina	241,476,432
5	Germany	648,450,175	Kazakhstan	241,222,161
6	Canada	643,470,359	Kenya	239,064,230
7	Mexico	157,059,158	Saudi Arabia	217,527,413
8	Estonia	95,391,163	Russian Federation	178,552,980
9	Switzerland	86,837,694	Mexico	155,317,847
10	Poland	85,866,992	Switzerland	151,998,017
**Rank**	**20** **21**		**202** **4**	
	**Country**	**Value**	**Country**	**Value**
1	USA	1,681,644,844	USA	3,622,093,039
2	Germany	843,939,212	Germany	1,230,628,570
3	Canada	761,802,056	Mexico	683,230,591
4	Mexico	459,423,377	Canada	505,754,041
5	Poland	393,197,933	Australia	451,554,801
6	Australia	344,231,884	Hungary	273,912,347
7	Kazakhstan	290,604,636	Austria	241,207,607
8	Austria	287,118,105	Slovakia	188,702,288
9	Russian Federation	237,504,644	Czechia	182,055,627
10	Czechia	162,852,579	Poland	174,181,815

**Table 6:** Top 10 countries by out-strength for selected years.

Rank	2013		2017	
	Country	Value	Country	Value
1	Ukraine	2,714,418,198	Mexico	2,153,773,765
2	Mexico	1,979,461,880	USA	1,074,984,665
3	China	1,335,205,060	China	740,264,160
4	USA	825,093,786	Slovakia	503,513,170
5	Slovakia	358,534,422	Russian Federation	500,299,106
6	Russian Federation	340,757,960	Poland	369,881,037
7	Poland	261,441,749	Romania	229,176,828
8	Germany	209,260,000	Ukraine	167,550,559
9	Romania	151,879,533	Germany	143,685,976
10	Bulgaria	122,626,357	Czechia	138,487,955
**Rank**	**20** **21**		**202** **4**	
	**Country**	**Value**	**Country**	**Value**
1	Mexico	1,598,799,717	Mexico	3,396,067,205
2	USA	1,109,293,659	USA	1,142,695,835
3	Slovakia	767,306,274	Slovakia	827,049,388
4	Russian Federation	601,487,196	China	761,651,770
5	China	537,157,006	Romania	515,753,575
6	Poland	277,165,158	Croatia	318,390,228
7	Bulgaria	265,196,303	Bulgaria	312,483,996
8	Romania	231,683,439	Poland	296,880,833
9	Canada	217,141,668	Canada	284,936,314
10	Belarus	187,621,799	Turkey	194,267,207

**Table 7:** Top 10 most critical source countries under full supply disruption.

Rank	0.1			0.3		
	Risky Source	Disrupted Scale	Iteration Time	Risky Source	Disrupted Scale	Iteration Time
1	Romania	114	6	Germany	107	7
2	Czechia	114	7	China	60	4
3	Germany	114	5	Japan	60	5
4	Switzerland	114	7	Romania	39	7
5	France	114	8	Serbia	37	6
6	Serbia	114	6	Croatia	36	6
7	Bulgaria	114	6	Slovakia	35	5
8	Poland	114	6	Bulgaria	20	4
9	Slovakia	114	5	Poland	12	3
10	Croatia	114	6	USA	11	5
**Rank**	**0.5**			**0.7**		
	**Risky Source**	**Disrupted Scale**	**Iteration Time**	**Risky Source**	**Disrupted Scale**	**Iteration Time**
1	Germany	66	5	China	53	4
2	China	56	4	Germany	9	3
3	USA	11	5	USA	7	3
4	Mexico	11	6	South Africa	7	2
5	Poland	10	3	Mexico	7	4
6	South Africa	7	2	Poland	6	3
7	Slovakia	7	4	Bulgaria	5	3
8	Russia	6	3	Russia	5	3
9	Belgium	5	2	Romania	4	2
10	Bulgaria	5	3	Netherlands	4	2

**Table 8:** Top 10 most critical source countries under full demand disruption.

Rank	0.1			0.3		
	Risky Source	Disrupted Scale	Iteration Time	Risky Source	Disrupted Scale	Iteration Time
1	USA	53	10	Austria	30	8
2	Canada	53	11	Germany	30	7
3	Mexico	53	11	USA	21	7
4	Philippines	48	9	Canada	21	8
5	Romania	47	9	Mexico	21	8
6	United Kingdom	47	9	Philippines	13	6
7	Austria	47	8	Singapore	11	6
8	Germany	47	7	Myanmar	10	5
9	Hungary	47	10	Spain	9	4
10	Switzerland	47	8	Azerbaijan	9	6
**Rank**	**0.5**			**0.7**		
	**Risky Source**	**Disrupted Scale**	**Iteration Time**	**Risky Source**	**Disrupted Scale**	**Iteration Time**
1	USA	17	7	USA	6	2
2	Mexico	17	8	Germany	6	3
3	Germany	13	3	Myanmar	6	5
4	Philippines	12	6	Spain	5	4
5	Myanmar	9	5	Netherlands	4	2
6	Spain	8	4	Guinea	4	4
7	Italy	7	3	Brazil	3	3
8	Latvia	5	4	Kazakhstan	3	3
9	Netherlands	4	2	Slovakia	3	3
10	Guinea	4	4	Panama	3	3

**Table 9:** Top 10 most critical trade edges under full cooperation disruption.

Rank	0.1			0.3		
	Risky Source	Disrupted Scale	Iteration Time	Risky Source	Disrupted Scale	Iteration Time
1	China → Australia	49	4	China → Australia	40	2
2	Bulgaria → Germany	37	6	Poland → Germany	7	3
3	Bulgaria → Poland	37	5	Mexico → USA	7	3
4	Romania → Germany	33	5	Russia → Uzbekistan	6	2
5	Romania → Hungary	33	5	Germany → Belgium	5	2
6	Romania → France	33	4	Belgium → Germany	5	2
7	Poland → UK	33	5	Spain → Myanmar	5	2
8	Poland → Germany	33	5	Slovakia → Spain	5	3
9	Slovakia → Czechia	27	4	Bulgaria → Germany	4	2
10	Slovakia → Germany	27	4	Ukraine → Lithuania	4	2

## Data Availability

The data used in this study were sourced from the United Nations Comtrade Database (UN Comtrade), which is publicly available at https://comtrade.un.org/. No new data were created or analyzed in this study.
